# Poly(lactic-co-glycolic Acid) Nanoparticle Encapsulated 17β-Estradiol Improves Spatial Memory and Increases Uterine Stimulation in Middle-Aged Ovariectomized Rats

**DOI:** 10.3389/fnbeh.2020.597690

**Published:** 2020-12-16

**Authors:** Alesia V. Prakapenka, Alicia M. Quihuis, Catherine G. Carson, Shruti Patel, Heather A. Bimonte-Nelson, Rachael W. Sirianni

**Affiliations:** ^1^Department of Psychology, Arizona State University, Tempe, AZ, United States; ^2^School of Life Sciences, Arizona State University, Tempe, AZ, United States; ^3^Arizona Alzheimer's Consortium, Phoenix, AZ, United States; ^4^Vivian L. Smith Department of Neurosurgery, University of Texas Health Science Center at Houston, Houston, TX, United States

**Keywords:** estrogen, PLGA, delivery, menopause, learning, memory

## Abstract

Hormone therapy that contains 17β-estradiol (E2) is used commonly for treatment of symptoms associated with menopause. E2 treatment has been shown to improve cognitive function following the decrease in ovarian hormones that is characteristic of menopause. However, once in circulation, the majority of E2 is bound to serum hormone binding globulin or albumin, becoming biologically inactive. Thus, therapeutic efficacy of E2 stands to benefit from increased bioavailability via sustained release of the hormone. Here, we focus on the encapsulation of E2 within polymeric nanoparticles composed of poly(lactic-co-glycolic) acid (PLGA). PLGA agent encapsulation offers several delivery advantages, including improved bioavailability and sustained biological activity of encapsulated agents. We hypothesized that delivery of E2 from PLGA nanoparticles would enhance the beneficial cognitive effects of E2 relative to free E2 or non-hormone loaded nanoparticle controls in a rat model of menopause. To test this hypothesis, spatial learning and memory were assessed in middle-aged ovariectomized rats receiving weekly subcutaneous treatment of either oil-control, free (oil-solubilized) E2, blank (non-hormone loaded) PLGA, or E2-loaded PLGA. Unexpectedly, learning and memory differed significantly between the two vehicle control groups. E2-loaded PLGA nanoparticles improved learning and memory relative to its control, while learning and memory were not different between free E2 and its vehicle control. These results suggest that delivery of E2 from PLGA nanoparticles offered cognitive benefit. However, when evaluating peripheral burden, E2-loaded PLGA was found to increase uterine stimulation compared to free E2, which is an undesired outcome, as estrogen exposure increases uterine cancer risk. In sum, a weekly E2 treatment regimen of E2 from PLGA nanoparticles increased cognitive efficacy and was accompanied with an adverse impact on the periphery, effects that may be due to the improved agent bioavailability and sustained biological activity offered by PLGA nanoparticle encapsulation. These findings underscore the risk of non-specific enhancement of E2 delivery and provide a basic framework for the study and development of E2's efficacy as a cognitive therapeutic with the aid of customizable polymeric nano-carriers.

## Introduction

The endogenous estrogen, 17β-estradiol (E2), is approved by U.S. Food and Drug Administration (FDA) for use in menopausal hormone therapy via oral, transdermal, and intravaginal routes of administration (U.S. FDA's Office of Women's Health, 2015). Orally administered E2 is susceptible to first-pass metabolism into less potent estrogen metabolites (e.g., estrone and estriol) as well as conversion to estrogen conjugates (e.g., E2-glucuronide, estrone-glucuronide, estrone-sulfate). Consequently, orally administered E2 yields relatively low E2 bioavailability, requiring daily administration to achieve desired therapeutic effects (Ruoff and Dziuk, 1994; Kuhl, 2005). Furthermore, the majority of circulating E2 becomes biologically inactive as it gets bound to serum hormone binding globulin or albumin (Anderson, 1974; Kuhl, 2005). Sustained delivery of E2 may offer some advantages. For example, transdermal application of E2, such as via a sustained release, bioadhesive skin patch, effectively bypasses first-pass metabolism and achieves therapeutic levels of E2 in circulation over a period of time ranging from days to weeks (Nachtigall, 1995; Buch et al., 1998; Samisoe, 2004). Both transdermal and oral administration routes can elicit cognitive benefits of hormone therapy in menopausal women (Wharton et al., 2011; Maki, 2012; Doty et al., 2015). Indeed, across multiple studies in women and in rodent models of menopause, E2 treatment following ovarian hormone loss can be cognitively beneficial (Sherwin, 2006; Maki, 2012; Mennenga and Bimonte-Nelson, 2013; Luine, 2014; Frick, 2015, 2020; Koebele and Bimonte-Nelson, 2015; Korol and Pisani, 2015; Prakapenka et al., 2018). Nevertheless, the 2017 North American Menopause Society (NAMS) statement does not recommend hormone therapy for prevention or treatment of cognitive decline due to lack of definitive findings as several clinical studies have found either neutral (e.g., Kronos Early Estrogen Prevention Study—Cognitive and Affective Study) or impairing (e.g., Women's Health Initiative Study of Cognitive Aging) cognitive effects of hormone therapy in menopausal women (Coker et al., 2010; Maki, 2012; Gleason et al., 2015; The NAMS 2017 Hormone Therapy Position Statement Advisory Panel, 2017). In women that have not had a hysterectomy, all E2-based hormone treatments must be accompanied by an opposing progestogen to offset undesired E2 exposure at the uterine tissue that would otherwise carry a cancer risk (The NAMS 2017 Hormone Therapy Position Statement Advisory Panel, 2017). The cognitively beneficial role of E2 in ovariectomized (Ovx) rodents is attenuated by the addition of progestogens, suggesting that progestogens can limit the therapeutic potential of E2 (Bimonte-Nelson et al., 2006; Harburger et al., 2007, 2009; Lowry et al., 2010; Prakapenka et al., 2018, 2020). Thus, although E2-containing hormone therapies are FDA-approved, safe, and efficacious, E2 delivery remains to be optimized.

Strategies for improving reproductive and brain health in women range from hormone chemical structure analyses to hormone delivery optimization and reproductive tissue engineering (Abdi et al., 2016; Amorim and Shikanov, 2016; Engler-Chiurazzi et al., 2017; Rivas Leonel et al., 2019). Our goal was to develop better E2 delivery approaches that will maximize the potential of hormone therapy in menopause. Here, we focused on poly(lactic-co-glycolic) acid (PLGA) nanoparticles as customizable drug carriers (Chasin and Langer, 1990; Danhier et al., 2012; Fazil et al., 2012; Cook et al., 2015; Householder et al., 2015; Mir et al., 2017; Prakapenka et al., 2017; Chung et al., 2020). PLGA is biocompatible, biodegradable, and FDA-approved for use in the clinic in both implanted and injectable forms (Chasin and Langer, 1990; Anderson and Shive, 1997; Danhier et al., 2012). Although several drawbacks of PLGA-based delivery systems have been identified, such as poor drug loading and limited *in vivo* characterization (Sharma et al., 2016), the ability to engineer various features of PLGA-based delivery systems, including particle size, surface charge, lactic acid to glycolic acid ratio, and agent encapsulation efficiency and release are significant advantages. Drugs that have been encapsulated within PLGA are released into aqueous environments in a sustained fashion (Lee and Pokorski, 2018). Sustained release confers advantages for enhancing bioavailability of poorly soluble agents, prolonging drug activity to enhance biological potency, and reducing unwanted toxicities that can result from traditional paradigms of periodic drug dosing (Chasin and Langer, 1990; Householder et al., 2015; Chen et al., 2017; Karthivashan et al., 2018). Indeed, E2 encapsulation in PLGA nanoparticles yields pharmacokinetic and pharmacodynamic profiles that are distinct, compared to free E2, and that are dependent on PLGA particle formulation (for review: Prakapenka et al., 2017). For instance, when E2-loaded PLGA particles are administered *in vivo*, levels of E2 in circulation are sustained. This yields greater total exposure of E2 to the body when delivered from a PLGA formulation compared to delivery in free form for several routes of administration (for review: Prakapenka et al., 2017). One of the major advantages of particle-based delivery is that the particle size and surface properties can be engineered to facilitate tissue-selective drug delivery (Makadia and Siegel, 2011; Kreuter, 2014; Mir et al., 2017; Prakapenka et al., 2017). For instance, surface-modification of E2 PLGA nanoparticles with Tween® 80 was found to significantly increase E2 levels in the brain relative to non-surface-modified E2 PLGA nanoparticles (Mittal et al., 2011). We have studied peptide strategies for achieving active targeting of hydrophobic molecules across the blood brain barrier (Cook et al., 2015; Chung et al., 2020); such a strategy could eventually be used to design a brain-selective hormone therapy.

Here, we hypothesized that delivery of E2 from PLGA nanoparticles would enhance cognition relative to free E2 or non-drug loaded nanoparticle controls. To test this hypothesis, we administered E2-loaded PLGA nanoparticles or freely solubilized E2 by subcutaneous injection to middle-aged rats that underwent Ovx as a surgically induced model of menopause. Rats received weekly subcutaneous oil-control, free (oil-solubilized) E2, blank (non-drug loaded) PLGA, or E2-loaded PLGA treatments, and were tested on a battery of behavioral tasks evaluating spatial learning and memory, including the water radial arm maze (WRAM) and Morris water maze (MWM). Uterine stimulation was assessed by measuring uterine horn weights as a marker of E2 peripheral burden. Unexpectedly, we observed a significant difference in spatial learning and memory for oil-control vs. blank PLGA groups. Additionally, although cognitive benefits were observed for delivery of E2 from PLGA compared to blank PLGA, delivery of E2 from PLGA was also observed to increase uterine stimulation compared to free E2. These observations raise major considerations for how formulations offering sustained release could impact cognition and cancer risk simultaneously. Altogether, our findings improve on the understanding of how the mode of E2 delivery impacts E2 function in a model of reproductive senescence and the associated hormonal decline, highlighting opportunities for the future design of hormone therapies.

## Materials and Methods

### Materials

All reagents for nanoparticle preparation and characterization, including dichloromethane (DCM), poly(vinyl alcohol) (PVA), methanol, phosphate buffered saline (PBS), and dimethyl sulfoxide (DMSO), were obtained from Sigma-Aldrich. 50:50 poly(DL-lactide-co-glycolide) (PLGA, ester terminated) was purchased from Durect Corporation (Cupertino, CA, USA). 17β-estradiol (E2) was purchased from Sigma-Aldrich.

### PLGA Nanoparticle Preparation

PLGA nanoparticles were prepared under endotoxin free conditions using a single-emulsion technique that was previously outlined by McCall and Sirianni (2013) but adapted to utilize PVA as the stabilizing agent. Briefly, to make blank PLGA nanoparticles (blank-PLGA), 200 mg of PLGA was dissolved in 2 mL of DCM:methanol (4:1) and added drop-wise to a 5% PVA aqueous phase on vortex, which created an emulsion. The emulsion was then ultrasonicated on ice 3 times in 10 s intervals (40% amplitude, Fisher Scientific Model 705 Sonic Dismembrator) and added to 84 mL of 0.3% PVA. Solvent was evaporated, which allowed nanoparticles to harden, over 3 h while stirring. Particles were then washed 3 times with endotoxin free water by centrifugation at 25,000 rcf for 20 min at 4°C (Beckman L8-80M Ultracentrifuge, F0630 rotor). Prior to lyophilization and storage (at −80°C), 75 mg of trehalose was added to the particles. To prepare E2-containing PLGA nanoparticles (E2-PLGA), 12 mg of E2 and 200 mg of PLGA were dissolved in 2 mL of DCM:methanol (4:1) and processed using the same approach as above.

### Nanoparticle Characterization

Size and morphology of blank-PLGA and E2-PLGA nanoparticles were assessed with scanning electron microscopy (SEM, FEI XL30) following the protocol described by McCall and Sirianni (2013). The average diameter of each nanoparticle batch was measured using ImageJ (National Institutes of Health). The hydrodynamic diameter and polydispersity for the nanoparticles was determined using dynamic light scatting (DLS, NanoBrook 90Plus Zeta particle analyzer, Brookhaven Instruments, Hotsville, NY).

To measure E2 loading, E2-PLGA nanoparticles were dissolved in DMSO at 1 mg/mL and the fluorescence of the sample was compared to a control curve constructed using blank-PLGA nanoparticles spiked with known E2 concentrations (280 nm/310 nm excitation/emission). E2 loading was then determined by dividing E2 concentration by PLGA concentration. A release profile for E2 was obtained by adding 10 mg/mL E2-PLGA in 1× PBS to Slide-A-Lyzer Dialysis Cassette (3,500 MWCO) and submerging the cassette in 4 L of 37°C 1× PBS release medium. For each time point, 20 μL of E2-PLGA sample were added to 180 μL of DMSO to obtain a 1 mg/mL concentration and the fluorescence of the samples was read in triplicate (280 nm/310 nm excitation/emission). The release medium was changed at 2.5, 7.5, and 21.5 h to ensure sink conditions.

### Animal Care

**Figure 1:**
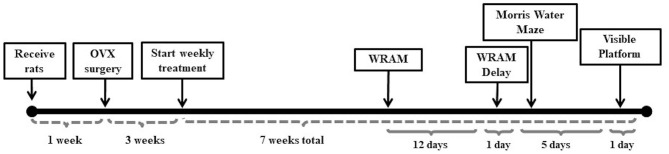
Study timeline. The study timeline illustrates the timing between Ovx surgery and initiation of weekly treatment injections, as well as between treatment initiation and behavior testing on the water radial-arm maze (WRAM), Morris water maze, and visible platform task.

Fischer-344 CDF 11-month old virgin female rats were ordered from the National Institute on Aging, Harlan Laboratories (Indianapolis, IN). Food and water were provided *ad libitum* and a 12-h light/dark cycle was imposed. Rats were pair-housed throughout the duration of the study. All procedures were approved by the Arizona State University IACUC and adhered to the standards set by the National Institutes of Health. Figure 1 depicts the timeline of experimental manipulations throughout the duration of the behavior study.

### Ovariectomy (Ovx)

Ovx surgery was performed on all rats under anesthesia using acute isoflurane inhalation as previously done (Mennenga et al., 2015c; Braden et al., 2017; Prakapenka et al., 2018). Briefly, rats received dorsolateral incisions to the skin and muscle, each ovary was removed following ligature to the tip of the uterine horn, and the skin and muscle were sutured closed. All rats received a subcutaneous injection of Rimadyl (5 mg/mL/kg) for pain prior to the start of surgery, as well as a subcutaneous injection of saline (2 mL) to prevent dehydration.

### Treatment Administration

Twenty-one days after Ovx, weekly subcutaneous treatment administration was initiated. Twenty rats were randomly assigned to receive 0.1 mL of either sesame oil (oil-control, *n* = 10) or 3 μg of free E2 (free E2, *n* = 10) in sesame oil, and twenty rats were randomly assigned to receive 0.1 mL of blank-PLGA (polymer weight matched to E2-PLGA, *n* = 10) suspended in saline or 3 μg of E2 encapsulated in PLGA (E2-PLGA, *n* = 10) suspended in saline. The oil-control and blank-PLGA treatments served as the vehicle controls in this study. E2 dose was based on prior work following a similar study timeline, which found a cognitively beneficial effect of daily free E2 (3 μg/day) relative to vehicle in Ovx middle-aged rats (Prakapenka et al., 2018). A weekly treatment regimen as opposed to daily treatment regimen was implemented to assess the effect of sustained E2 delivery from PLGA nanoparticles on spatial learning and memory.

### Behavioral Assessment of Cognitive Function

#### Water Radial-Arm Maze (WRAM)

Beginning on the day of the fourth treatment administration, all animals were tested on the win-shift WRAM task for 13 days to evaluate spatial working and reference memory (Bimonte and Denenberg, 1999; Bimonte-Nelson, 2015c; Mennenga and Bimonte-Nelson, 2015; Mennenga et al., 2015c; Braden et al., 2017). The maze was located in a room that was set up with abundant spatial cues to aid in spatial navigation. This maze contained 8 arms, 38.1 × 12.7 cm dimensions for each arm, and was filled with water made opaque with non-toxic paint, kept at 18–20°C. Hidden platforms (10 cm in diameter) were submerged in 4 out of the 8 arms. A pre-determined set of platform locations, which were kept fixed throughout all 13 days of testing, were randomly assigned to each animal. Each animal was allowed 3 min per trial to find a platform. The trial started when the animal was dropped off at the start arm location and the trial ended when the animal either found or was led to the platform (after 3 min passed). Animals were kept on the platform for 15 s, after which they were placed in a heated testing cage. During the 30 s inter-trial interval (ITI), the just-found platform was taken out of the maze and the water was cleaned with a fishnet to remove debris and redistribute any olfactory cues; the next trial was started at the end of the ITI. In this manner, there was one trial per platform (4 trials per day), resulting in increased strain on the memory system with each additional found platform that was removed, leading to an increase in working memory load as trials progress. A 6-h delay was implemented between trials 2 and 3 on the last day of testing, day 13, to assess delayed memory retention. To examine performance on the WRAM, error arm entries were recorded and scored on orthogonal measures of reference and working memory. A reference memory (RM) error was defined as the first entry, within a day, into a non-platformed arm. A working memory incorrect (WMI) error was defined as re-entry, within a day, into a non-platformed arm. A working memory correct (WMC) error was defined as any entry, within a day, into an arm that was previously platformed.

#### Morris Water Maze (MWM)

MWM testing started the day following completion of WRAM testing to examine spatial reference memory performance, and lasted for 5 days (Talboom et al., 2008, 2014; Bimonte-Nelson, 2015a). The maze was a 188 cm in diameter circular tub located in a room filled with spatial cues to aid in spatial navigation. One platform (10 cm in diameter) was submerged in 18–20°C water, made opaque with non-toxic paint, in the northeast quadrant of the tub. The platform location was kept consistent for all trials across each day of testing. There were four starting locations (north, south, east, or west), one per trial with a total of 4 trials per day. The trial started when an animal was dropped off at one of these locations and the trial was completed when the animal either found the submerged platform or was led to the platform after the maximum trial time of 60 s. Each animal was kept on the platform for 15 s and then returned back to a heated cage. The ITI was 5–8 min. The Ethovision tracking system (Noldus Instruments, Wageningen, The Netherlands) was used to determine each animal's swim distance. A probe trial was added on the last day of testing as an additional 5th trial to examine spatial localization during which the platform was removed and each animal's swim distance in each quadrant during a total of 60 s was measured.

#### Visible Platform

The 1-day visible platform task, initiated the day after MWM testing, was used to evaluate the motor and visual ability of each animal to complete a water-escape maze task (Bimonte-Nelson, 2015b; Mennenga et al., 2015a,c). The maze was a 100 cm x 60 cm rectangular tub that was filled with 18–20°C clear water. One black platform (10 cm in diameter) was placed in the tub so that the top of the platform was 4 cm above the surface of the water. All obvious spatial cues surrounding the maze were blocked using a matted white curtain. Each animal was allowed 90 s per trial to find the platform, the location of which varied semi-randomly between trials, with a total of 6 trials and an ITI of 5–8 min. The trial started when an animal was dropped off from a set location and the trial ended when the animal either found the platform or was led to the platform after 90 s. Each animal was given 15 s on the platform before being placed into a heated testing cage.

### Confirming Systemic Presence of E2

#### Blood Serum Analysis

Double antibody liquid-phase radioimmunoassay (Beckman Coulter, Brea, CA), performed by the Core Endocrinology Laboratory of the Pennsylvania State University, College of Medicine, was used to determine circulating E2 and estrone, a metabolite of E2, levels in blood serum (Engler-Chiurazzi et al., 2012; Mennenga et al., 2015c; Koebele et al., 2017; Prakapenka et al., 2018). On the day of the 7th treatment injection, blood was collected via cardiocentesis following anesthesia with isoflurane at least 1 h after treatment injection in the same order as behavior testing occurred. To obtain serum, blood was allowed to clot at 4°C (Vacutainer 367986, Becton Dickinson and Company, Franklin Lakes, NJ, USA) and then centrifuged for 20 min at 3,000 rpm at 4°C. Serum was stored at −20°C until radioimmunoassay analysis. E2-specific antibodies were used with ^125^I-labeled E2 as the tracer for the E2 assay with a functional sensitivity of 4 pg/ml. Inter-assay coefficients of variation at a mean level of 6 pg/ml E2 averaged 8%. Estrone-specific antibodies were used with ^125^I-labeled estrone as the tracer for the estrone assay with a functional sensitivity of 16 pg/ml. Inter-assay coefficients of variation for estrone at a mean level of 90 pg/ml averaged 11%.

#### Uterine Horn Weights

Exposure to circulating estrogens increases uterine horn weight in rodents (Westerlind et al., 1998; Engler-Chiurazzi et al., 2012; Mennenga et al., 2015b; Prakapenka et al., 2018). To obtain uterine horn weight, uterine horns were removed and trimmed of visible fat at sacrifice; the wet weight of uterine horns was then used to confirm Ovx and to assess E2 exposure.

### Statistical Analyses

Repeated measures ANOVA was run separately to test for a main effect of Day and Treatment x Day interaction on the WRAM and on the MWM to determine learning. For the WRAM, each memory measure (WMC, WMI, and RM errors) was evaluated across days 1–12 of testing. For the MWM, Total Swim Distance was evaluated across days 1–5 of testing. Additionally, for the probe trial, each treatment group was analyzed separately using a repeated measures ANOVA to evaluate percent swim distance in the previously platformed quadrant (northeast) compared to percent swim distance in the directly opposite quadrant (southwest). For the visible platform, a repeated measures ANOVA was run to test for a main effect of Trial, a main effect of Treatment, and a Trial x Treatment interaction to assess learning and the ability to perform a water-escape task for all treatment groups.

For WRAM treatment effects, WRAM testing was split into three blocks, Block 1 (days 2–5) to represent the early acquisition phase, Block 2 (days 6–9) to represent the late acquisition phase, and Block 3 (days 10–12) to represent the asymptotic phase (Koebele et al., 2019). *A priori* comparison repeated measures ANOVA was run for each Block to directly compare oil-control and blank-PLGA treatments to evaluate vehicle effects. Block 3 did in fact exhibit an effect of Vehicle, and Vehicle x Trial interaction, for WMC and WMI measures, discussed in detail in the results section. Due to the Vehicle effect, additional analyses included *a priori* comparisons to only compare free E2 to its respective vehicle control (oil-control) and E2-PLGA to its respective vehicle control (blank-PLGA) for WMC, WMI, and RM errors made on each Block of testing. *A priori* comparisons between free E2 and E2-PLGA groups could not be made due to the Vehicle effect (Denenberg, 1996). In the case of a significant Treatment x Trial interaction, Trial 3 and Trial 4 were analyzed separately to evaluate performance on the moderate and high working memory load trials, respectively. For all analyses, Treatment was the independent variable and Trials nested within Days were the repeated measures. For the WRAM delay, errors made on the WMC, WMI, and RM memory measures on the post-delay trial, Trial 3 on Day 13, were compared to the baseline trial, Trial 3 on Day 12, within each treatment group as was done previously (Braden et al., 2017). For MWM, *a priori* comparisons were done to analyze Total Swim Distance for free E2 vs. oil-control, as well as E2-PLGA vs. blank-PLGA, across the 5 days of testing. Treatment was set as the independent variable and Trials nested within Days were set as the repeated measures.

Planned comparison one-way ANOVAs were used to separately analyze E2 and estrone blood serum levels and uterine horn weights. The blank-PLGA group was compared to the oil-control group (vehicle effect), the free E2 group compared to the oil-control group, the E2-PLGA group compared to the blank-PLGA group, and the E2-PLGA group compared to the free E2 group. Treatment was set as the independent variable and E2 Level, Estrone Level, and Uterine Horn Weight were set as the dependent variables.

For all analyses, the significance level for alpha was set at *p* < 0.05. Nanoparticle characterization measurements were expressed as mean ± standard deviation; behavior measurements, hormone serum levels, and uterine horn weights were expressed as mean ± standard error of the mean (s.e.m.).

## Results

### Nanoparticle Characterization

**Figure 2:**
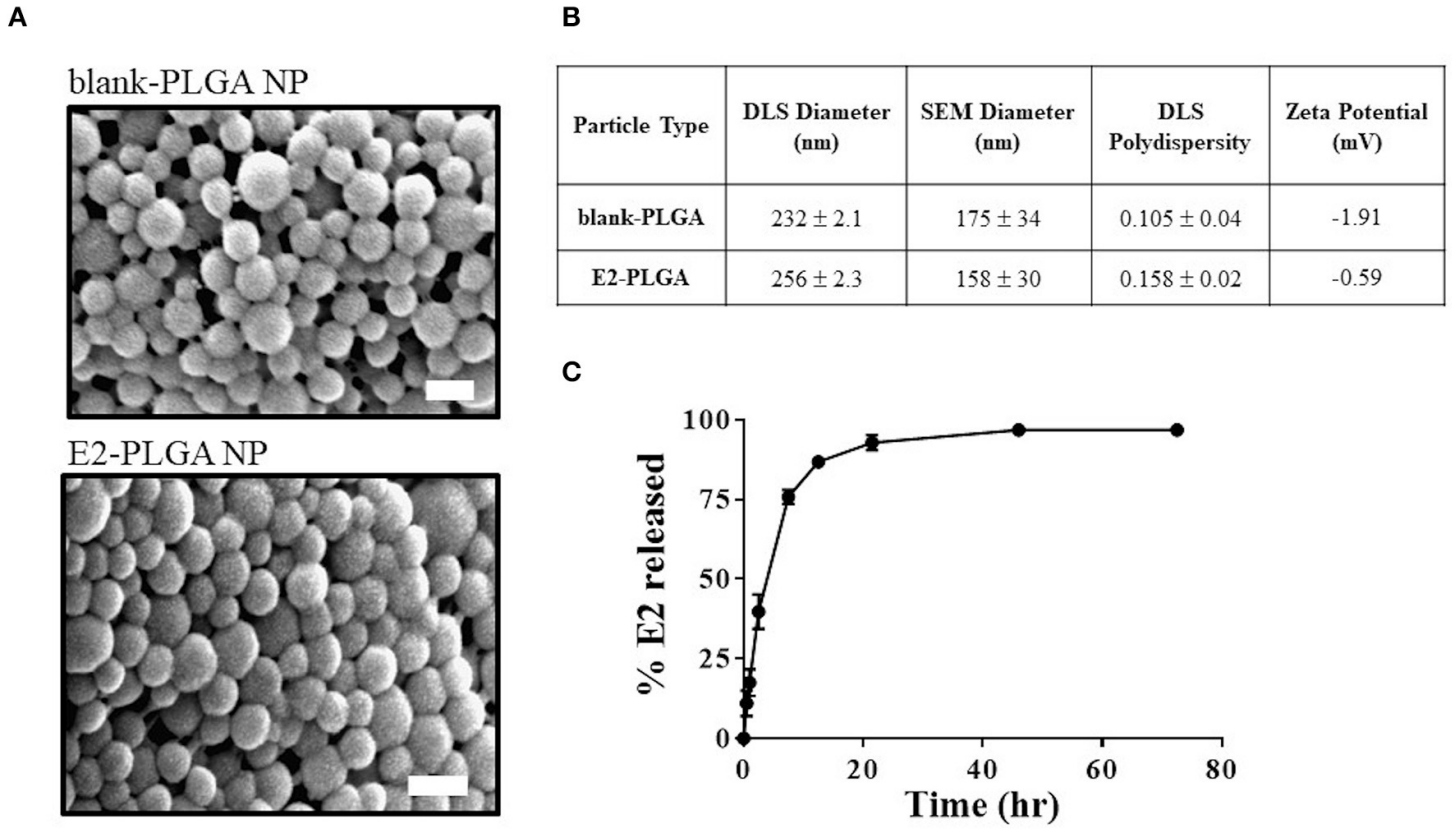
Blank-PLGA and E2-PLGA characterization. **(A)** Representative SEM images of blank-PLGA and E2-PLGA nanoparticles; scale bar is 200 nm. **(B)** E2 release profile from PLGA nanoparticles, *n* = 4 per time point. **(C)** Table summarizing the size, polydispersity, and zeta potential of each nanoparticle formulation. Data are presented as mean ± standard deviation.

E2 loading in PLGA nanoparticles was 4.45% weight to weight (w/w). SEM image analysis revealed that the blank-PLGA and E2-PLGA nanoparticles were spherical with an average diameter of 175 ± 34 nm and 158 ± 30 nm, respectively (Figure 2A). DLS analysis yielded the average hydrodynamic diameter for blank-PLGA and E2-PLGA nanoparticles of 232 ± 2.1 nm and 256 ± 2.3 nm, respectively. The polydispersity index for blank-PLGA and E2-PLGA nanoparticles was 0.105 ± 0.04 and 0.158 ± 0.02, respectively (Figure 2B). The surface charge for both batches of nanoparticles was close to neutral, measuring −1.91 mV for blank-PLGA and −0.59 mV for E2-PLGA (Figure 2B). PLGA nanoparticles exhibited rapid release of E2 within the first 8 h (~80% of E2 released) followed by a slow sustained release. Complete release of E2 was observed after 48 h of incubation (Figure 2C).

### Behavioral Assessment of Cognitive Function

#### Water Radial-Arm Maze (WRAM)

**Figure 3:**
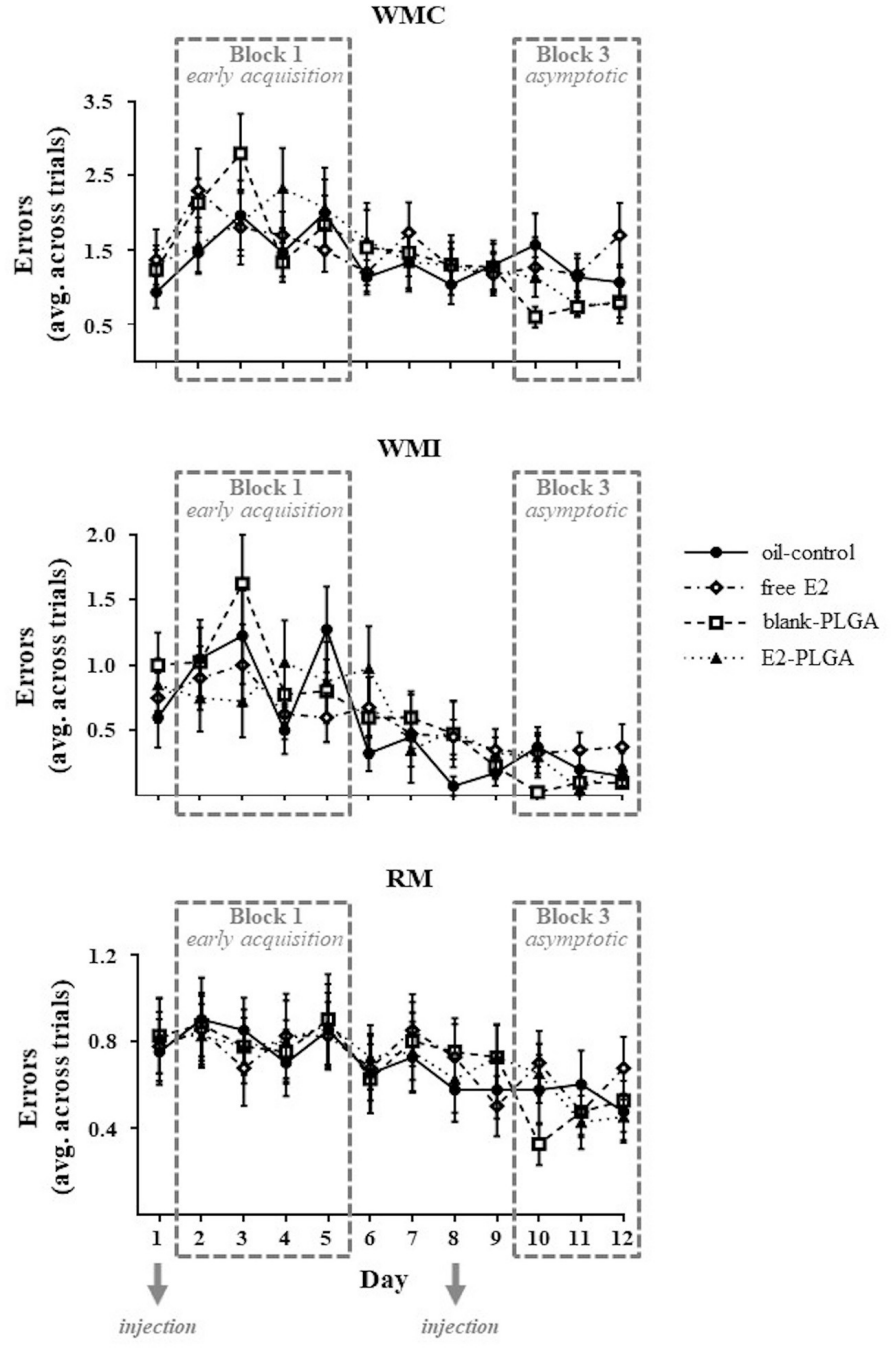
Water radial-arm maze learning curves for each memory measure. WMC, WMI, and RM errors made on each day of testing, collapsed across trials, are shown to visualize the learning curve for each memory measure and treatment group. The days of weekly injection administration are noted. Data are presented as mean ± s.e.m.

The WRAM measures spatial working and spatial reference memory. For each memory measure, there was a main effect of Day, indicating learning of the WRAM as illustrated by a decrease in WMC [*F*_(11, 36)_ = 5.676, *p* < 0.0001; Figure 3], WMI [*F*_(11, 36)_ = 9.840, *p* < 0.0001; Figure 3], and RM [*F*_(11, 36)_ = 7.816, *p* < 0.0001; Figure 3] errors across all days (days 1–12) of non-delay testing. There were no significant Treatment x Day interactions for each memory measure, demonstrating that WRAM learning for all days of testing did not differ based on treatment.

**Figure 4:**
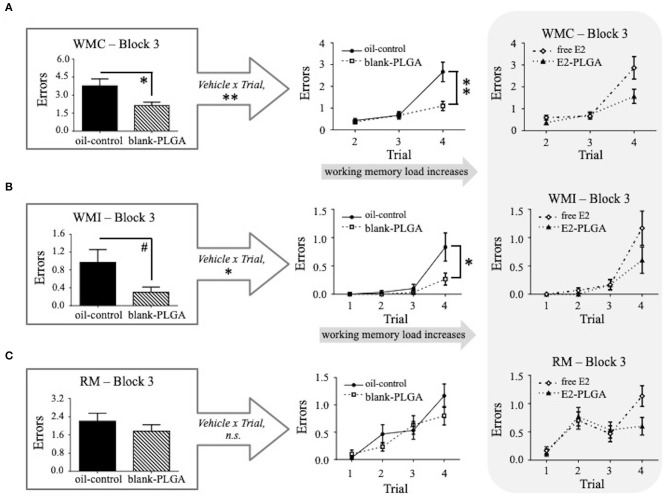
Water radial-arm maze: vehicle effect on the asymptotic phase. **(A)** WMC errors on Block 3 of testing, the asymptotic phase, where the blank-PLGA made fewer WMC errors than the oil-control group. The vehicle by trial graph illustrates that the blank-PLGA made fewer WMC errors than oil-control on Trial 4, the highest working memory load trial. **(B)** WMI errors on Block 3 of testing where the blank-PLGA tended to make fewer WMI errors than the oil-control group. The vehicle by trial graph illustrates that the blank-PLGA made fewer WMI errors than oil-control on Trial 4, the highest working memory load trial. **(C)** RM errors on Block 3 of testing, where no differences between the two vehicle control groups were observed. Treatment by trial graphs within the gray box in **(A–C)** show WMC, WMI, and RM errors made on Block 3 of testing for free E2 and E2-PLGA groups. Data are presented as mean ± s.e.m. **p < 0.01, *p < 0.05, #*p* < 0.1.

Performance on Blocks 1, 2, and 3 of the WRAM was evaluated between the two vehicle control groups (oil-control and blank-PLGA) across the three memory measures: WMC, WMI, and RM. No main effects of Vehicle or Vehicle x Trial interactions were seen for WMC, WMI, or RM errors made on Block 1, the early acquisition phase during which the rules of the task were being learned, or Block 2, the late acquisition phase during which rats continue to learn but overall make less errors in relation to early acquisition phase. However, results for the WMC measure on Block 3, the asymptotic phase evaluating memory retention, revealed a significant main effect of Vehicle [*F*_(1, 18)_ = 8.170, *p* < 0.05; Figure 4A] and a Vehicle x Trial interaction [*F*_(2, 36)_ = 7.803, *p* < 0.01; Figure 4A], where the oil-control group made more errors than the blank-PLGA group. Further analyses indicated that this Vehicle effect was particularly pronounced on Trial 4 (*p* < 0.01), the highest working memory load trial evaluated on this version of the WRAM. For the WMI measure on Block 3, there was a marginal effect of Vehicle [*F*_(1, 18)_ = 4.296, *p* = 0.05; Figure 4B] and a significant Vehicle x Trial interaction [*F*_(3, 54)_ = 4.056, *p* < 0.05; Figure 4B], where the oil-control group tended to make more errors than the blank-PLGA group. This effect was significant on the highest working memory load trial, Trial 4 (*p* < 0.05). There was no significant effect of Vehicle and no Vehicle x Trial interaction for the RM measure on Block 3 (Figure 4C). Thus, the two control vehicles exhibited significantly different effects on spatial working memory on Block 3, the asymptotic phase of the WRAM when rules of the task should be learned.

**Figure 5:**
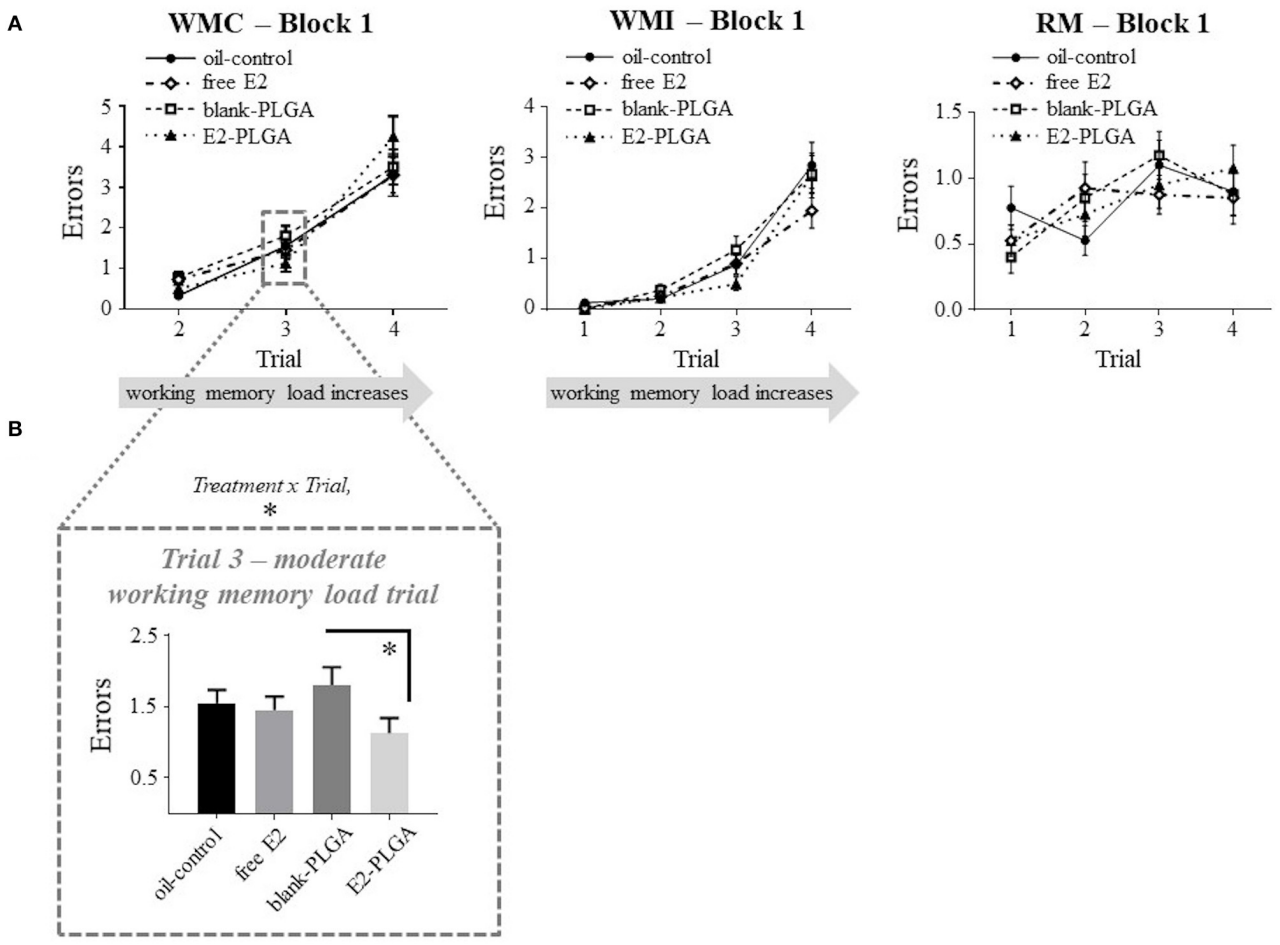
Water radial-arm maze: early acquisition phase. **(A)** Treatment by trial graphs for WMC, WMI, and RM errors made on Block 1, the early acquisition phase. For WMC, there was a treatment x trial interaction between blank-PLGA and E2-PLGA groups, and further analyses revealed that on Trial 3, the moderate working memory load trial, E2-PLGA made fewer WMC errors than blank-PLGA, shown in **(B)**. Data are presented as mean ± s.e.m. *p < 0.05.

Following the analyses of vehicle effects, *a priori* comparisons were performed to evaluate E2-PLGA vs. its vehicle (blank-PLGA) as well as free E2 vs. its vehicle (oil-control). For the E2-PLGA and blank-PLGA comparison, there was no main effect of Treatment, and a significant Treatment x Trial interaction, for WMC errors on Block 1 of testing [*F*_(2, 36)_ = 4.486, *p* < 0.05; Figure 5A]. The E2-PLGA group made fewer WMC errors on Trial 3 (*p* < 0.05; Figure 5B), the moderate working memory load trial, and did not significantly differ on WMC errors made on Trial 4, the high working memory load trial, compared to the blank-PLGA group. There were no Treatment differences and no Treatment x Trial interactions on Blocks 2 or 3 for the WMC measure (data not shown), as well as Blocks 1, 2, or 3 for WMI or RM measures, between E2-PLGA and blank-PLGA groups (see Figure 5A for Block 1 Treatment x Trial interactions). There were no Treatment differences and no Treatment x Trial interactions on Blocks 1, 2, or 3 for WMC, WMI, or RM measures between free E2 and oil-control groups. Taken together, a main effect of vehicle was observed where the blank PLGA nanoparticle group improved spatial working memory compared to the oil-control group. Additionally, these data suggest that the E2-PLGA treatment did enhance spatial working memory compared to its blank-PLGA control during the early acquisition phase (Block 1) of the WRAM, but only when working memory load was moderate.

**Figure 6:**
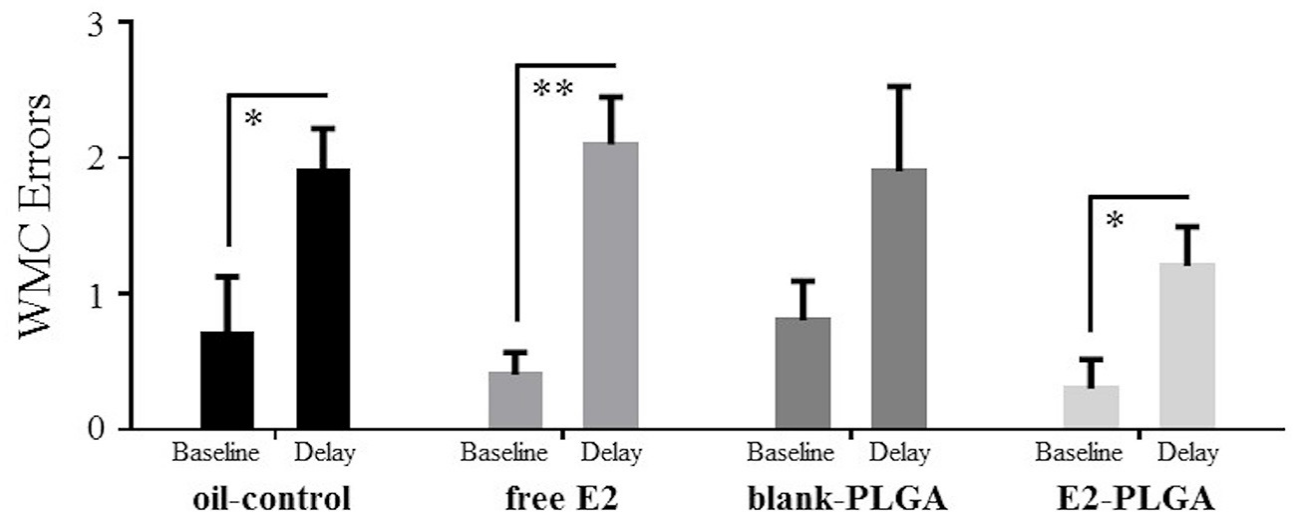
WMC errors made after a 6-h delay on the water radial-arm maze. WMC errors made on the Delay trial, Trial 3 on Day 13, and WMC errors made on the Baseline trial, Trial 3 on Day 12, for each treatment group. Oil-control, free E2, and E2-PLGA groups made more WMC errors on the Delay trial relative to the Baseline trial, suggesting delay-induced impairment. Differences in WMC errors made on the Delay trial relative to the Baseline trial by the blank-PLGA group did not reach statistical significance. Data are presented as mean ± s.e.m. **p < 0.01, *p < 0.05.

On day 13 of the WRAM, a 6-h delay was implemented between Trials 2 and 3. Errors made on the baseline trial, Trial 3 on Day 12, were compared to errors made on the post-delay trial, Trial 3 on Day 13, within each treatment group. For WMC errors, oil-control [*F*_(1, 9)_ = 6.612, *p* < 0.05; Figure 6], free E2 [*F*_(1, 9)_ = 18.447, *p* < 0.01; Figure 6], and E2-PLGA [*F*_(1, 9)_ = 6.688, *p* < 0.05; Figure 6] treatment groups made more errors on the delay trial compared to the baseline trial, indicating delay-induced impairment. The blank-PLGA group tended to make more WMC errors on the delay trial compared to the baseline trial, although this did not reach statistical significance [*F*_(1, 9)_ = 3.120, ns; Figure 6]. For WMI and RM errors, there were no differences within each treatment group between errors made on the delay trial compared to the baseline trial (data not shown).

#### Morris Water Maze (MWM)

**Figure 7:**
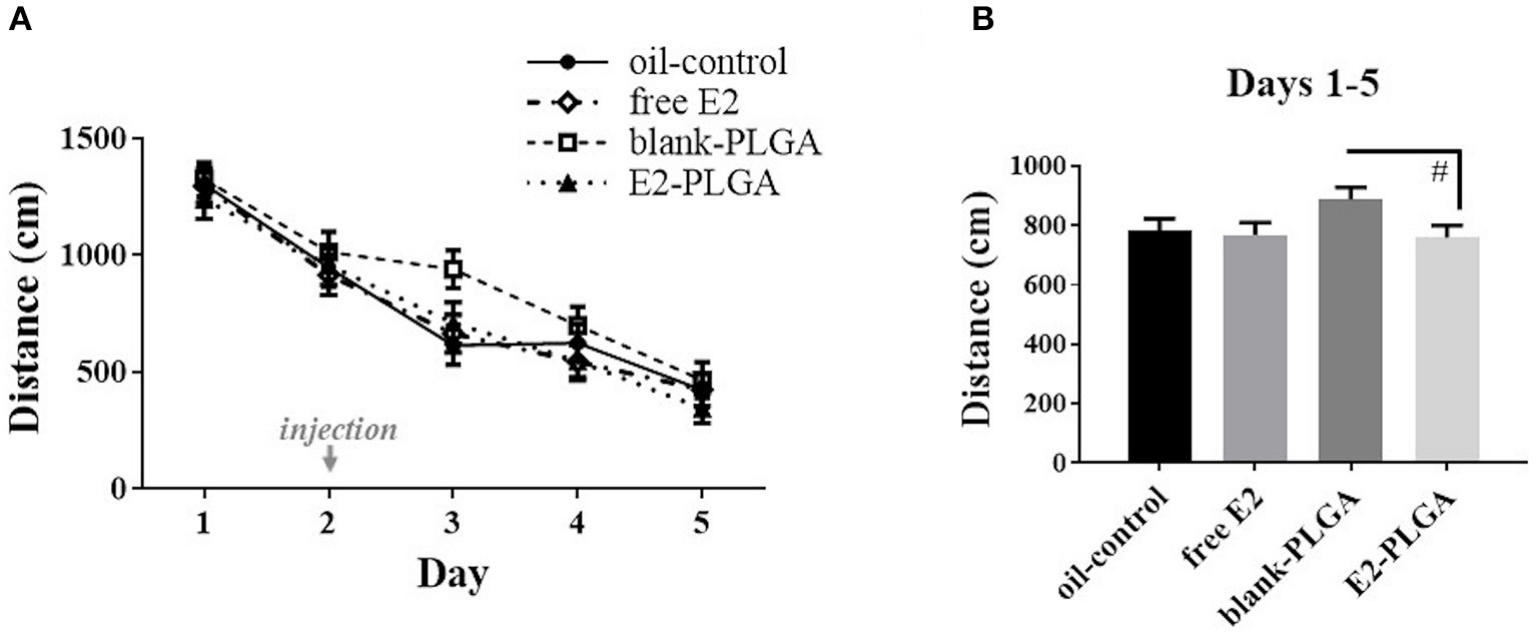
Morris water maze performance. **(A)** Distance to platform for each day of testing, collapsed across trials, depicting learning of the task by each treatment group. The day of treatment injection is noted on the x-axis. **(B)** Distance to platform collapsed across days and trials, noting that the E2-PLGA group tended to swim a shorter distance to platform than the blank-PLGA group. Data are presented as mean ± s.e.m. #*p* < 0.1.

Analysis of spatial reference memory performance across the 5 days of testing on the MWM revealed a main effect of Day, with decreasing Total Swim Distance scores across days [*F*_(4, 36)_ = 78.929, *p* < 0.0001; Figure 7A]. The four treatment groups had a similar learning profile across days as the Treatment x Day effect was not significant. *A priori* comparisons revealed a marginal effect of Treatment [*F*_(1, 18)_ = 3.181, *p* < 0.1; Figure 7B], and no Treatment x Day interaction, across the 5 days of testing between the blank-PLGA group and the E2-PLGA group, suggesting that there was a trend for the E2-PLGA group to swim a shorter distance to the platform than the blank-PLGA group collapsed across all testing days. There was no effect of Treatment and no Treatment x Day interaction across the 5 days of testing between the oil-control and free E2 groups. For the probe trial, oil-control [*F*_(1, 9)_ = 61.563, *p* < 0.0001], free E2 [*F*_(1, 9)_ = 14.357, *p* < 0.01], blank-PLGA [*F*_(1, 9)_ = 69.291, *p* < 0.0001], and E2-PLGA [*F*_(1, 9)_ = 108.477, *p* < 0.0001] groups each swam a greater percent distance in the target northeast quadrant where the platform was previously located compared to the opposite southwest quadrant that never contained the platform, suggesting that each treatment group was able to spatially localize to the platform location (data not shown).

#### Visible Platform

The visible platform task was used to confirm visual and motor capability to complete a water-escape maze task. Across all six trials, there was a main effect of Trial [*F*_(5, 36)_ = 3.225, *p* < 0.01], and the average latency to platform for all subjects across the six trials was 8.9 s (data not shown). There was no significant Treatment x Trial interaction and a marginal Treatment effect for all six trials [*F*_(3, 36)_ = 2.658, *p* < 0.1]. No Treatment effect was seen for the last three trials tested, suggesting that all groups exhibited similar ability to perform on a water-escape maze task, especially by the latter half of testing.

### Confirming Systemic Presence of E2

#### Blood Serum Analysis

**Figure 8:**
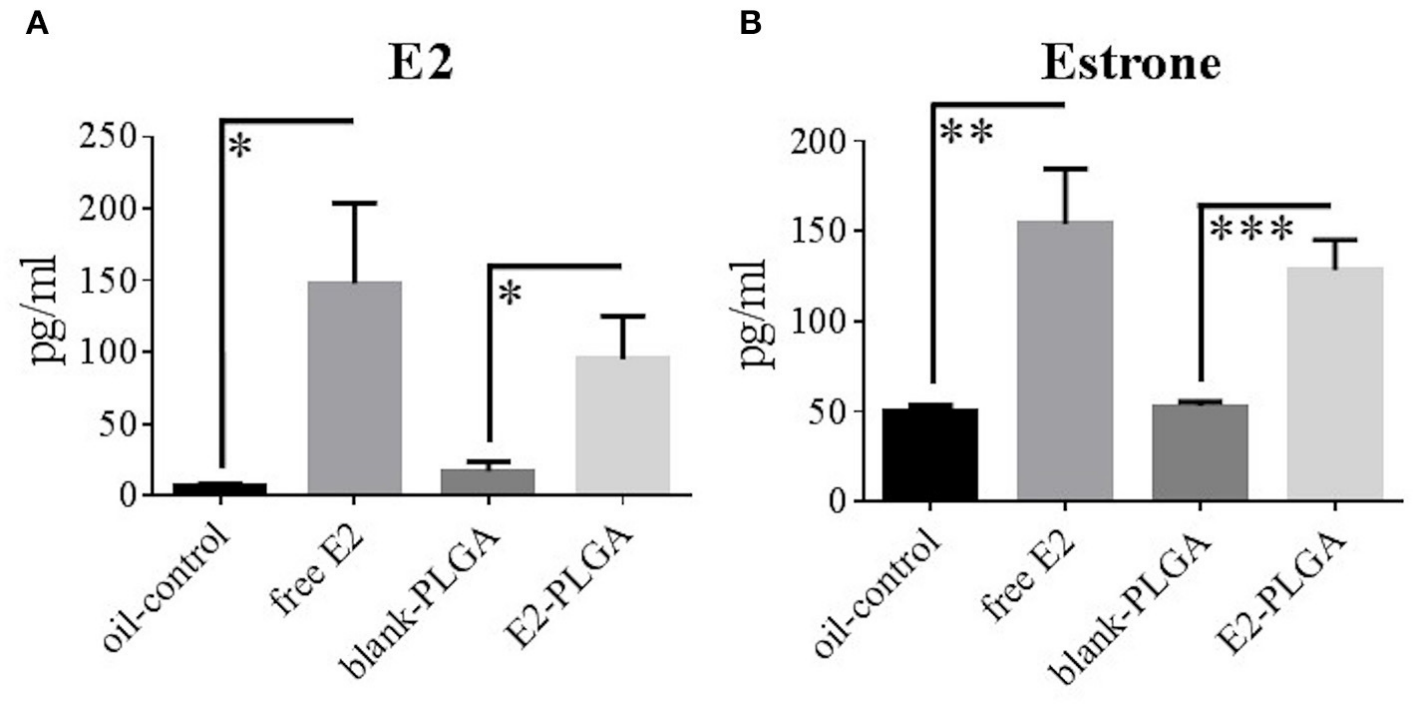
E2 and estrone blood serum levels. **(A)** E2 blood serum levels collected following the 7th treatment injection, with free E2 and E2-PLGA exhibiting greater E2 levels than oil-control and blank-PLGA, respectively. **(B)** Estrone blood serum levels collected following the 7th treatment injection, with free E2 and E2-PLGA exhibiting greater estrone levels than oil-control and blank-PLGA, respectively. Data are presented as mean ± s.e.m. ***p < 0.001, **p < 0.01, *p < 0.05.

Blood serum levels of E2 and estrone were obtained to determine circulating levels of free E2 and its ability to be metabolized into estrone following weekly subcutaneous administration. Specifically, blood serum was collected in order of behavior testing the day of the 7th weekly treatment administration. There were no Treatment effects between the blank-PLGA and oil-control vehicle groups in E2 levels or differences in estrone levels, indicating that the two vehicle controls did not differentially impact circulating E2 and estrone levels. There was a main effect of Treatment for E2 levels between blank-PLGA and E2-PLGA groups [*F*_(1, 18)_ = 6.475, *p* < 0.05; Figure 8A] as well as a main effect of Treatment for E2 levels between oil-control and free E2 groups [*F*_(1, 18)_ = 6.316, *p* < 0.05; Figure 8A], where the E2-PLGA and free E2 groups each had higher E2 levels than their respective controls. In regularly cycling, ovary-intact rats, reported average E2 levels can range from as low as 17 pg/ml to as high as 144 pg/ml across the estrous cycle (Mennenga and Bimonte-Nelson, 2015). Thus, the mean ± s.e.m. E2 circulating levels from free E2 (148 ± 56 pg/ml) and E2-PLGA (95 ± 30 pg/ml) groups are representative of high physiological E2 levels of an ovary-intact rat. Additionally, there was a main effect of Treatment for estrone levels between blank-PLGA and E2-PLGA groups [*F*_(1, 18)_ = 20.451, *p* < 0.001; Figure 8B] and a main effect of Treatment for estrone levels between oil-control and free E2 groups [*F*_(1, 18)_ = 11.153, *p* < 0.01; Figure 8B], where the E2-PLGA and free E2 groups each had higher estrone levels than their respective controls. There were no Treatment effects between the E2-PLGA and free E2 groups in E2 levels or in estrone levels, suggesting that there was no difference in circulating free E2, or in its metabolism to estrone, between free vs. nanoparticle encapsulated weekly subcutaneous administration of E2.

#### Uterine Horn Weight

**Figure 9:**
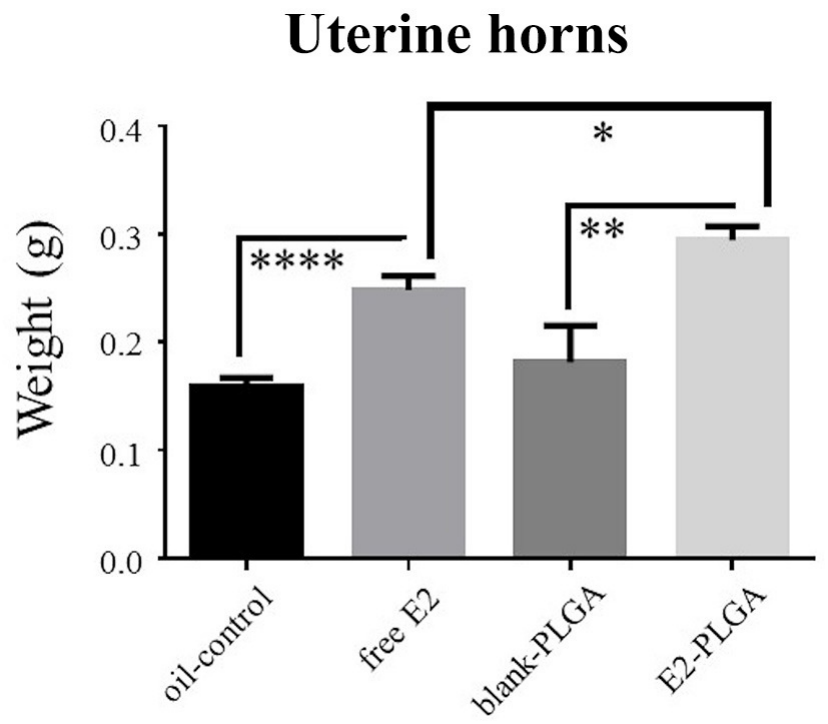
Uterine horn weights. Free E2 and E2-PLGA treatments increased uterine horn weight relative to oil-control and blank-PLGA, respectively, indicating E2-induced uterine stimulation. E2-PLGA had greater uterine horn weight compared to free E2, suggesting greater E2 exposure with the E2-PLGA treatment formulation. Data are presented as mean ± s.e.m. ****p < 0.0001, **p < 0.01, *p < 0.05.

Uterine horn weights were obtained the same day as blood serum as a marker for uterine stimulation across treatments. There was no Treatment effect between the blank-PLGA and oil-control vehicle groups, indicating that the two vehicle controls did not differentially impact uterine horn weight. There was a main effect of Treatment between the blank-PLGA and E2-PLGA groups [*F*_(1, 18)_ = 9.52, *p* < 0.01; Figure 9] as well as a main effect of Treatment between oil-control and free E2 groups [*F*_(1, 18)_ = 31.458, *p* < 0.0001; Figure 9], where the E2-PLGA and free E2 groups each had higher uterine horn weights than their respective control groups. Interestingly, there was also a main effect of Treatment between the E2-PLGA and free E2 groups [*F*_(1, 18)_ = 6.230, *p* < 0.05; Figure 9], where the E2-PLGA group had higher uterine horn weights than the free E2 group, indicating that E2 encapsulated in nanoparticles resulted in greater uterine horn stimulation than free E2.

## Discussion

The present study aimed to evaluate the cognitive, circulating hormone, and uterine effects of weekly E2 PLGA nanoparticle treatment relative to weekly free E2 and weekly blank PLGA nanoparticle treatments in middle-aged, Ovx rats. E2 encapsulation in PLGA nanoparticles enhanced spatial working memory on the WRAM and trended toward enhancing spatial reference memory on the MWM compared to blank PLGA nanoparticles. Interestingly, there was a vehicle effect, whereby the blank PLGA nanoparticle group made fewer errors than the oil-control group on the asymptotic phase of the WRAM. Consequently, due to differences in cognition as a function of the vehicle, E2 PLGA nanoparticle vs. free E2 treatment effects on learning and memory could not be directly evaluated in the present study. It is important to note that all groups performed similarly on the control visible platform task, indicating that all rats exhibited visual and motor capability to complete water-escape tasks. When peripheral exposure to E2 was assessed, we found no difference in vehicle effects on uterine horn weight between the oil-control and blank PLGA nanoparticle groups. Both E2-treated groups exhibited increased uterine horn weight compared to their respective vehicle control, and E2 PLGA nanoparticle treatment had greater uterine horn weight than free E2 treatment, indicating potentially increased or sustained exposure of E2 at the uterine horns with nanoparticle encapsulation.

The WRAM is often used to evaluate hormone effects on spatial working and reference memory in middle-aged, Ovx rats (Acosta et al., 2010; Braden et al., 2017; Prakapenka et al., 2018). In the present study, all rats learned the task and their learning trajectories across the 12 days of testing did not differ by treatment. WRAM performance was further evaluated across the early acquisition phase (Block 1, days 2–5), the late acquisition phase (Block 2, days 6–9), and the asymptotic phase (Block 3, days 10–12) of testing. A vehicle effect was observed on the asymptotic phase evaluating spatial memory retention. This effect revealed that blank PLGA nanoparticle treatment, the vehicle for E2 PLGA nanoparticle treatment, resulted in fewer WMC and WMI errors committed as compared to the oil-control treatment, the vehicle for free E2 treatment. The vehicle effect was primarily driven by performance on Trial 4, when working memory demand was highest. Following a 6-h delay, analyses revealed that all treatment groups exhibited delay-induced impairment on the WMC measure, although the blank PLGA treatment group did not reach statistical significance on the delay trial compared to the baseline trial, further supporting vehicle-specific differences on spatial working memory. The vehicle effect was surprising because the blank PLGA nanoparticles, suspended in sterile saline, were not anticipated to impact spatial learning and memory differently from the sesame oil, and both vehicle groups were expected to exhibit poor cognitive performance since Ovx generally impairs spatial learning and memory (Bimonte and Denenberg, 1999; Markowska and Savonenko, 2002; Feng et al., 2004; Gibbs and Johnson, 2008; Talboom et al., 2008). In prior behavior studies, saline and sesame oil groups were typically combined as one control group for treatment comparisons because the saline and sesame oil effects were found to not differ on the spatial working and reference memory food-motivated radial arm maze (Tarbali and Khezri, 2016) and on the spatial reference memory MWM (Abdulla et al., 1993). Studies evaluating drug-encapsulated PLGA nano- and micro- particle effects on learning and memory that included both saline as well as blank particle control groups revealed no vehicle effects on cognitive performance on the passive-avoidance task (Wang et al., 2007; Khalin et al., 2016). Nevertheless, it is plausible that the PLGA characteristics contribute to the distinct vehicle effects on spatial memory evidenced in the present study. Following systemic administration, PLGA degrades into lactic acid and glycolic acid, and recent studies suggest that lactic acid plays a critical role in long-term memory formation, spatial working memory, as well as neuroplasticity (Newman et al., 2011; Suzuki et al., 2011; Yang et al., 2014; Sun et al., 2017). Although the total quantity of lactic acid produced by PLGA nanoparticle degradation in this circumstance is expected to be small, we cannot exclude this as a possibility. Additionally, the immune system is involved in maintaining spatial learning and memory (Ziv et al., 2006; Yirmiya and Goshen, 2011). Since PLGA nanoparticles may elicit a foreign body immune response following administration (Fournier et al., 2003; Semete et al., 2010), the evidenced vehicle effect could in part be mediated by the immune response. Further, stress response as measured by elevations in corticosterone levels can be elicited in response to treatment administration, such as by oral gavage, and depends on vehicle type (Brown et al., 2000). Thus, the present study underscores the importance of incorporating proper controls and statistical analyses when evaluating novel delivery systems, especially in the context of behavioral outcomes. Indeed, the addition of a seemingly inert carrier can influence learning and memory outcomes on its own. Deeper investigation into the cellular and biochemical sources of these effects will be an important avenue for future work.

Due to the exhibited vehicle effects, WRAM and MWM performance was further evaluated by comparing E2 PLGA treatment relative to its control, as well as comparing free E2 treatment relative to its control. This allowed us to determine the effects of weekly E2 PLGA treatment, and the effects of weekly free E2 treatment, on spatial learning and memory. On the early acquisition block of WRAM testing—when rats were learning the rules of the task—a trial by treatment interaction revealed that E2 PLGA nanoparticle treatment resulted in fewer WMC errors relative to blank PLGA nanoparticle treatment on Trial 3, when working memory load was moderate. These findings are consistent with literature, and support a cognitively beneficial role of E2 following a decrease in circulating levels of ovarian-derived hormones (Daniel et al., 1997, 2006; Luine et al., 1998; Bimonte and Denenberg, 1999; Fader et al., 1999; Gibbs and Johnson, 2008; Rodgers et al., 2010; Prakapenka et al., 2018). The free E2 treatment did not differ from its control on any of the three memory measures evaluated across the three blocks of WRAM testing. For the MWM, all treatment groups learned the task and exhibited a similar learning trajectory across testing days. Additionally, probe trial evaluations revealed that all treatment groups spatially localized to the platform location. There was a trend for the E2 encapsulated PLGA treatment group to swim a shorter distance to the platform across the 5 days of testing, indicating better performance relative to its control, although the effect did not reach statistical significance. The free E2 treatment did not differ in swim distance to the platform relative to its control. Thus, overall findings from the present study indicate that E2 encapsulation in PLGA nanoparticles enhanced the hormone's beneficial cognitive effects when a weekly subcutaneous treatment regimen was implemented. Specifically, E2 PLGA improved spatial memory compared to its control whereas free E2 did not differ from its control in middle-aged, Ovx rats.

The overarching goal of optimizing the delivery of E2 for cognitive therapy is to enhance E2-mediated memory effects while minimizing exposure of the hormone to peripheral tissue. Following E2 PLGA and free E2 treatments, circulating levels of E2 and of estrone, a metabolite of E2, were higher relative to each treatment's respective controls, confirming the presence of E2 within a high physiological range (Mennenga and Bimonte-Nelson, 2015) as well as the presence of its metabolite in the periphery. E2 and estrone circulating levels did not differ between E2 PLGA and free E2 treatment groups, indicating that E2 encapsulation in PLGA nanoparticles did not impact circulating levels of E2 or of the hormone's metabolism to estrone. Additionally, both E2 PLGA and free E2 increased uterine horn weight compared to their respective controls, confirming E2-induced uterine exposure and stimulation; uterine weight measurements are a noted confirmation of estrogen milieu, with estrogen exposure expected to increase uterine horn weights in Ovx rats (Westerlind et al., 1998; Prakapenka et al., 2018). Further, E2 PLGA increased uterine horn weight compared to free E2, suggesting prolonged or increased exposure of the uterine horns to E2 following its delivery when encapsulated in PLGA nanoparticles, an effect most likely due to the sustained release of the hormone from PLGA. Taken together, delivery of E2 encapsulated in PLGA nanoparticles improved spatial memory and yielded increased E2 uterine stimulating effects relative to free E2 delivery. Full pharmacokinetic profiling of E2 delivery as a function of formulation would be an interesting avenue for future work. It would also be relevant to study whether lower total dosing of E2 could achieve therapeutic effects for a PLGA-based system compared to free E2, which could be one way to reduce otherwise negative impact of peripheral E2 exposure.

In sum, despite an apparent effect of vehicle on spatial working memory, the implementation of PLGA nanoparticles for E2 delivery enhanced the cognitively beneficial effects of the hormone. Indeed, weekly E2 PLGA treatment resulted in improved spatial working memory on the WRAM task and showed a trend in improved spatial reference memory on the MWM task relative to control treatment, whereas free E2 treatment did not impact spatial learning and memory relative to control treatment. E2 PLGA treatment also resulted in heightened effects of the hormone on uterine stimulation relative to free E2, suggesting that the use of PLGA nanoparticles, at least in a non-modified (non-targeted) formulation, is not ideal for uterine protection within the current treatment parameters. Given that uterine protection is an important component of a safe and effective hormone therapy profile, this outcome should be considered when rating risk to benefit ratios of hormone therapy utilization. Altogether, the established baseline of cognitive and uterine effects following E2 PLGA treatment vs. free E2 treatment provided within this study sets the stage for future work focused on developing nanocarrier systems capable of sustaining E2 bioavailability in the brain while reducing peripheral exposure. Future strategies will focus on modifying the methods outlined in the present study, such as through the implementation of an alternate route of administration, evaluation of dose-dependent effects, tissue-specific targeting, or changes to the nanocarrier formulation.

An important outcome of this work is that we show that when implementing novel drug delivery platforms or novel outcome measures for drug delivery platforms, the evaluation of vehicle controls is critical and can impact interpretation of results as the carrier itself may exhibit subtle effects on nuanced behavioral measures. In consideration of improving E2 treatment for potential use as a cognitive therapy, alternate drug delivery strategies must also be evaluated, such as the addition of a specific targeting moiety, route of administration, or use of other drug carrier types. Undeniably, the merging of behavioral neuroendocrinology with biomedical engineering is an exciting, and still novel, avenue that can yield hormone therapy options optimized for desired therapeutic efficacy, such as treatment of a specific symptom with minimized peripheral burden, and will ultimately lead to improved quality of life for women across their lifespan.

## Data Availability Statement

The raw data supporting the conclusions of this article will be made available by the authors, without undue reservation.

## Ethics Statement

The animal study was reviewed and approved by Arizona State University Institutional Animal Care and Use Committee (IACUC).

## Author Contributions

AP, HB-N, and RS were responsible for conception and design of the study, data analysis and interpretation, and writing of the manuscript. AP, AQ, CC, and SP contributed to data collection. All authors edited and approved the manuscript.

## Conflict of Interest

The authors declare that the research was conducted in the absence of any commercial or financial relationships that could be construed as a potential conflict of interest.
